# Vascular endothelial growth factor signaling requires glycine to promote angiogenesis

**DOI:** 10.1038/s41598-017-15246-3

**Published:** 2017-11-07

**Authors:** Dongqing Guo, Colin E. Murdoch, Hao Xu, Hui Shi, Dayue Darrel Duan, Asif Ahmed, Yuchun Gu

**Affiliations:** 1 School of Life Sciences, Beijing University of Chinese Medicine, Beijing, 100029 China; 2 Aston Medical Research Institute, Aston Medical School, Aston University, Birmingham, B4 7ET UK; 3 Molecular Pharmacology Laboratory, Institute of Molecular Medicine, Peking University, Beijing, 100871 China; 4 Laboratory of Cardiovascular Phenomics, Department of Pharmacology, Centre for Cardiovascular Research, Centre for Molecular Medicine, University of Nevada School of Medicine, Reno, Nevada 89557-0318 USA; 5 Novolife Regenerative Medicine Centre, Aston Medical Research Institute, Aston Medical School, Aston University, Birmingham, B4 7ET UK

## Abstract

Peripheral vascular occlusive disease (PVOD) is a common manifestation of atherosclerosis, and it has a high rate of morbidity. Therapeutic angiogenesis would re-establish blood perfusion and rescue ischemic tissue. Vascular endothelial growth factor (VEGF) induces angiogenesis and can potentially be used to treat ischemic diseases, yet in clinical trials VEGF has not fulfilled its full potential with side effects. Whether amino acids promote angiogenesis and the molecular mechanisms are largely unknown. Here we showed that (1) Glycine significantly promoted angiogenesis both *in vitro* and *in vivo* and effectively protected mitochondrial function. (2) Activation of glycine transporter 1(GlyT1) induced by VEGF led to an increase in intracellular glycine. (3) Glycine directly bounded to voltage dependent anion channel 1 (VDAC1) on the mitochondrial outer membrane and inhibited its opening. These original results highlight glycine as a necessary mediator in VEGF signalling via the GlyT1-glycine-mTOR-VDAC1 axis pathway. Therefore, the findings in this study are of significance providing new mechanistic insights into angiogenesis and providing better understanding of glycine function in angiogenesis, which may provide valuable information for development of novel therapeutic targets for the treatment of angiogenic vascular disorders.

## Introduction

Peripheral vascular occlusive disease (PVOD) is a common manifestation of atherosclerosis, and it has a high rate of morbidity^1^. Sufficient collateral vessel formation can potentially re-establish blood perfusion and rescue ischemic tissue. Angiogenesis is the generation of new blood vessels from existing vasculature^2^, which is vital for reproduction, development, and tissue repair^3^. It is also a major pathological process of many angiogenic disorders such as malignancies^4^, diabetic retinopathy^5^, rheumatoid arthritis^6^, psoriasis^7^ and rosacea^8^. VEGF is a key regulator of angiogenesis^9^ and the only specific mitogen for endothelial cells^10^. A better understanding of the VEGF-mediated signaling pathways in endothelial cells will help determine the key molecular targets on angiogenesis in the vasculature. To date, three key pathways in the VEGF signals have been reported which include Raf-MEK (mitogen-activated or extracellular signal-regulated protein kinase kinase)-MAPK (mitogen-activated protein kinase) pathway, PI3K (phosphatidylinositol 3-kinase)-Akt pathway, and Src-FAK (focal adhesion kinase) pathway^11^. Transcriptional coactivator PGC-1 alpha (peroxisome-proliferator-activated receptor-gamma coactivator-1 alpha), a potent metabolic sensor and regulator, is induced by a lack of nutrients and oxygen. PGC-1 alpha powerfully regulates *in vivo* VEGF expression and angiogenesis in cultured muscle cells and skeletal muscle^12^. To identify additional signals in the pathogenesis of human vascular disorders, a large-scale proteomic approach was applied to define the mammalian target of rapamycin complex 2 (mTORC2)-fork head box protein O1 (FoxO1) axis in VEGF signal and feedback activation of receptor tyrosine kinases^13^. In addition to stimulating endothelial cell proliferation and migration, VEGF is known to cause vasodilation and to increase vascular permeability^14^. All these actions contribute to the pathophysiological processes related to VEGF over-activity. So it is very important to find new and safe targets which modulate angiogenesis. Amino acids have been identified as potential candidates in promoting survival^15,16^, but the effects of amino acids in the angiogenesis are still unknown.

Here we investigated the key mediators involved in VEGF signaling and found that glycine is required in the VEGF signaling pathways. Our data provide insights that glycine could promote the mitochondrial function by inhibiting VDAC1 opening.

## Results

### Glycine was required in the VEGF signals

**Figure 1:**
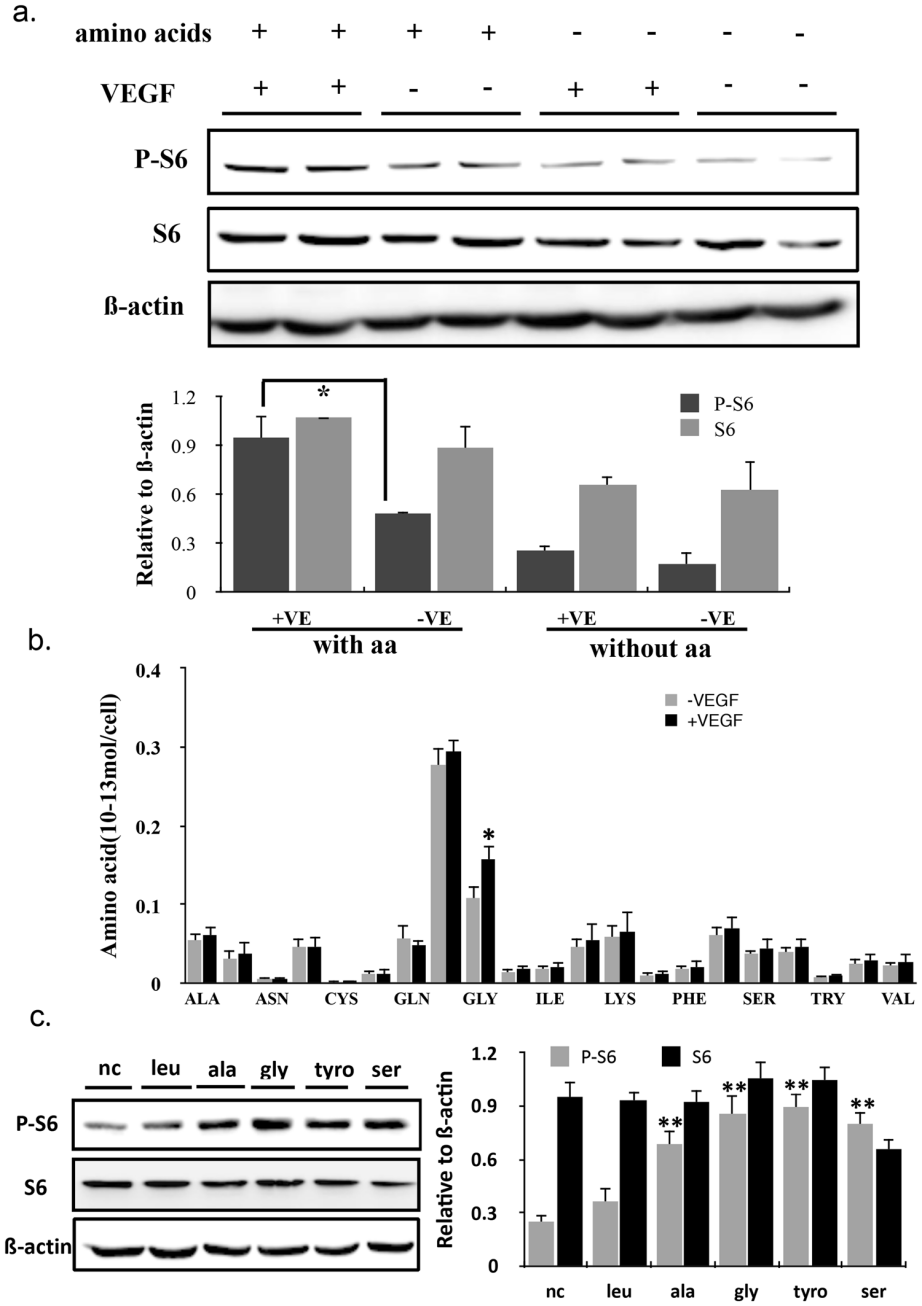
Glycine was required in the VEGF signals. (**a**) HUVECs cultured in the medium with or without amino acids were treated with 20 ng/ml VEGF for 24 hours. Immunoblots of P-S6 and S6 were shown. Membranes were reprobed for ß-actin as loading control. Gray values were analyzed by the Image J software and presented in the graph (n = 3). (**b**) Intracellular content of alanine(Ala), arginine(Arg), asparagine(Asn), aspartic acid(Asp), cysteine(Cys), γ-aminobutyric acid(Gaba), glutamine(Gln), glutamic acid(Glu), glycine(Gly), histidine (His), isoleucine(Ile), leucine(Leu), lysine 37, methionine(Met), phenylalanine(Phe), proline(Pro), serine(Ser), threonine(Thr), tryptophan(Trp), tyrosine(Tyr) and valine(Val) in control and 20 ng/ml VEGF-treated ECs for 24 h. LC-MS was used to detect relative intracellular quantities of amino acids in ECs in the absence and presence of VEGF (n = 3). (**c**) Immunoblot of total S6 and P-S6 in untreated and different amino acids-treated ECs. The concentration of amino acids: glycine(140 uM), leu(1.33 mM), tyro(1 mM), ala(1 mM), ser(1 mM). The treatment time was 5 h. Quantitative analysis was presented in the graph (n = 3).

VEGF promotes endothelial cell migration, proliferation, angiogenesis and entering of cell cycle. mTOR is a central regulator of cellular growth^17^. VEGF induces phosphorylation of the well characterised ribosomal S6 protein, while has no effect on mTOR expression. Removal of all amino acids in the cell medium also resulted in loss of VEGF-induced ECs proliferation with no change of the expression of P-S6 in response to VEGF (Fig. 1a). Taken together, these results suggested a unique role for amino acids in the mTOR signaling induced by VEGF.

To find out which amino acids involved in the VEGF signals, liquid chromatography-mass spectrometry (LC-MS) was used and revealed that glycine was higher in ECs cytoplasm induced by VEGF (Fig. 1b). Our results showed that exogenous glycine obviously elevated the level of P-S6 via the mTOR signaling pathway, but had no effect on S6 expression and other amino acids also have the same effects (Fig. 1c).

### Glycine promoted the angiogenesis

**Figure 2:**
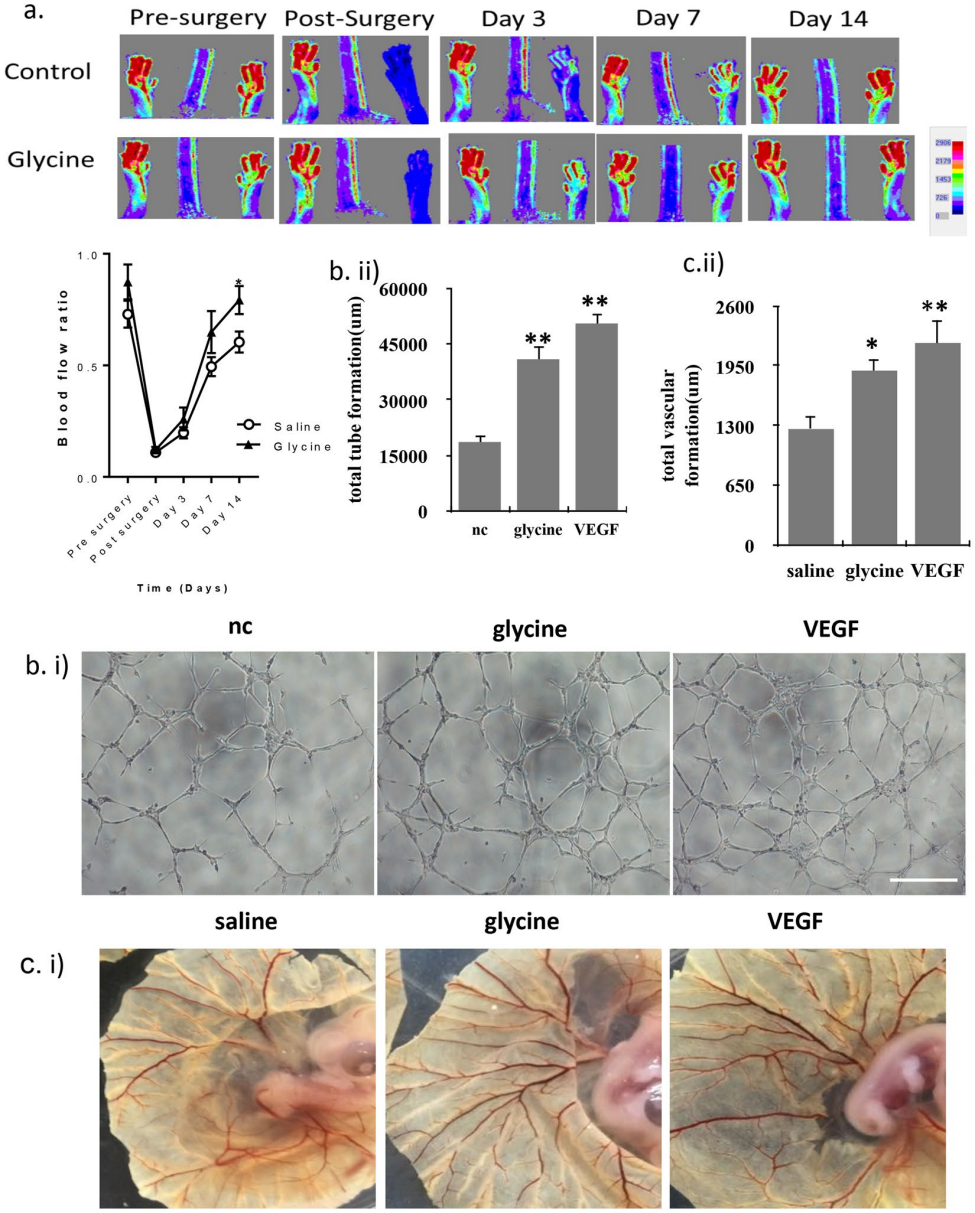
Glycine could promote the angiogenesis. (**a**) Hind-limb ischemia was created in C57 mice through femoral artery ligation. Upper: Serial laser Doppler analysis of blood perfusion in hindlimbs of mice injected with saline, glycine or VEGF at the day 3, 7 and 14. The concentration: 140 uM glycine and 20 ng/ml VEGF. The volume: 100 ul. Down: Quantitative analysis of blood flow using percentage of the ischemic limb relative to the control limb (n = 6). Results represented mean ± SEM of n independent experiments. **P < 0.01, *P < 0.05, 1-way ANOVA. (**b**) Representative images of *in vitro* tube formation in control, 140uM glycine or 20 ng/ml VEGF-treated ECs. Total sprouting length was indicated in the graph. Scale bar, 500 um (n = 5). (**c**) Representative images of the angiogenesis of saline, 140 uM glycine or 20 ng/ml VEGF-treated chorioallantoic membrane (CAM) (n = 6).

Next we explored the role of glycine *in vivo* using the hindlimb ischemia model. During the hind limb ischemia surgery glycine was administered into the saphenous artery and immediately tied off prior to a 1.5 mm section being removed. The control mice were treated with same volume of saline. Continuous administration of glycine through dietary supplementation after ischemia was used to maintain glycine therapeutic effects during the two-week period. Glycine treatment enhanced neovascularization significantly promoting the recovery of vascular flow from the third day (Fig. 2a). Glycine promoted HUVECs to form networks *in vitro* (Fig. 2b). Using an *in vivo* assay, glycine alone promoted the angiogenesis on the chorioallantoic membrane (Fig. 2c). During these assays, VEGF was used as positive control.

### Glycine obviously promoted the mitochondrial function

**Figure 3:**
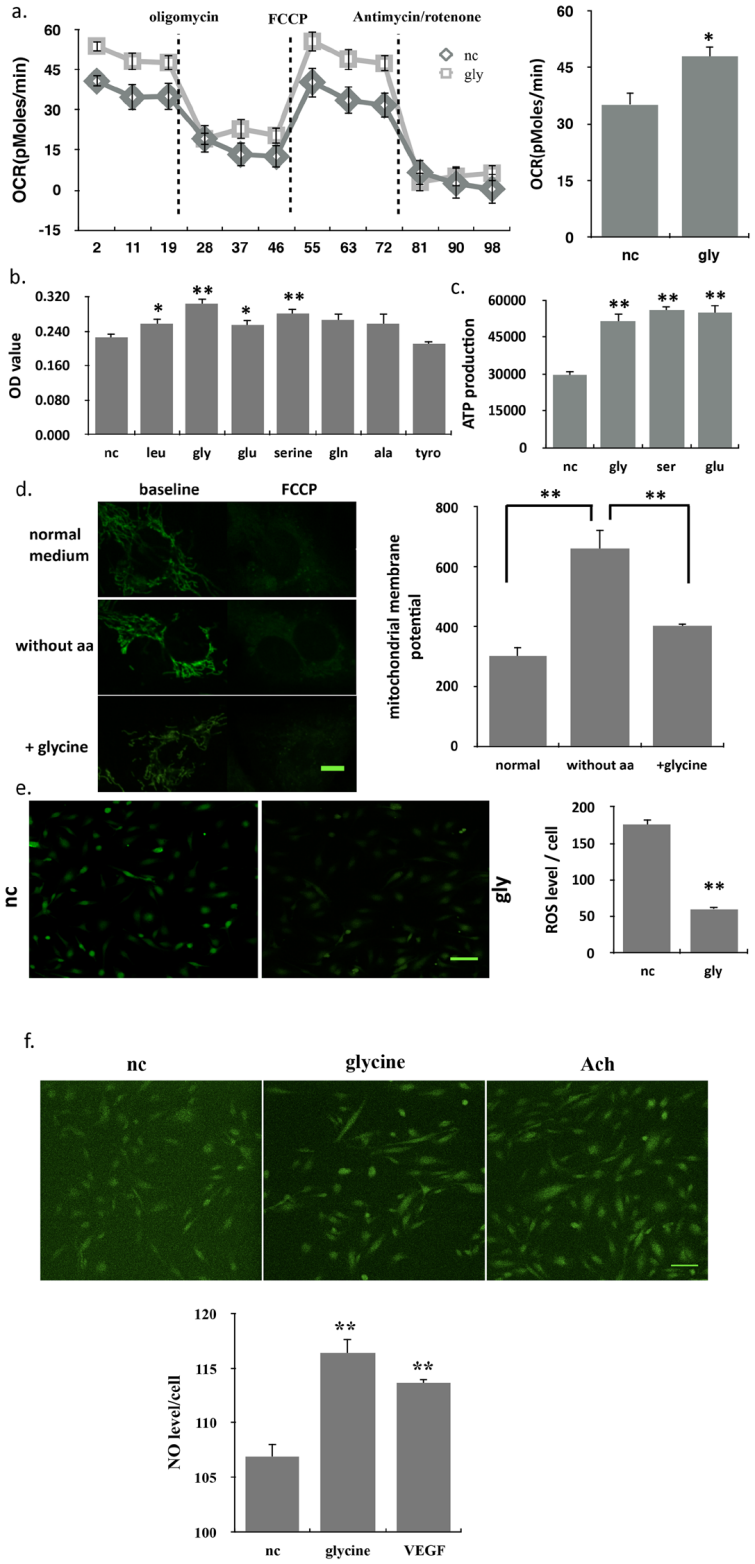
Glycine obviously promoted the mitochondrial function. (**a**) FCCP coupled oxygen consumption rate (OCR) showing higher respiration in ECs treated with glycine than controls (n = 10). (**b**) Mitochondrial activity was tested by MTT in ECs treated with different amino acids (n = 12). (**c**) Intracellular ATP levels in control and glycine-treated ECs (n = 16). (**d**) Changes in mitochondrial membrane potential (ΔΨm) in ECs cultured in RPMI-1640 without amino acids medium or adding glycine. ΔΨm was indicated by Rh-123 fluorescence staining (λex = 460, λem = 520) and observed with a laser confocal microscope. Scale bar, 10 µm (n = 16). (**e**) Intracellular ROS levels in control and glycine-treated ECs as measured using DCFH-DA. ROS staining was observed with a laser confocal microscope. Left: representative images of ROS staining; right: ROS fluorescence intensity in per cell. Scale bar, 100 µm (n = 10). All values represented mean ± SEM of n independent experiments. **P < 0.01, *P < 0.05, 1-way ANOVA. (**f**) Intracellular NO levels in control, glycine or Ach-treated ECs as measured using DAF-FM DA. NO staining was observed with a laser confocal microscope. Upper: representative images of NO staining; Down: NO fluorescence intensity in per cell. Scale bar, 100 µm (n = 10). All values represented mean ± SEM of n independent experiments. **P < 0.01, *P < 0.05, 1-way ANOVA.

We explored whether glycine regulated angiogenesis by affecting the mitochondrial functions. Interestingly, glycine induced increase of oxygen consumption (OCR) coupled to FCCP (Fig. 3a). The mitochondrial activity also significantly increased with the stimulation of glycine (Fig. 3b). Intracellular ATP production increased for nearly 100% in glycine-induced ECs (Fig. 3c). Mitochondrial membrane potential (ΔΨm) is essential to maintain mitochondrial oxidative phosphorylation, produces the adenosine triphosphate and maintains the normal physiological function of cells^18^. When ECs cultured in the medium without amino acids, the ΔΨm increased significantly in contrast to the group with completed medium, while the group with supplement of glycine maintained the ΔΨm stability (Fig. 3d). Moreover, glycine alone decreased the intracellular ROS production (Fig. 3e) and promoted the nitric oxide production (Fig. 3f).

### VEGF activated GlyT1 and glycine directly bound to VDAC1 on the mitochondria

**Figure 4:**
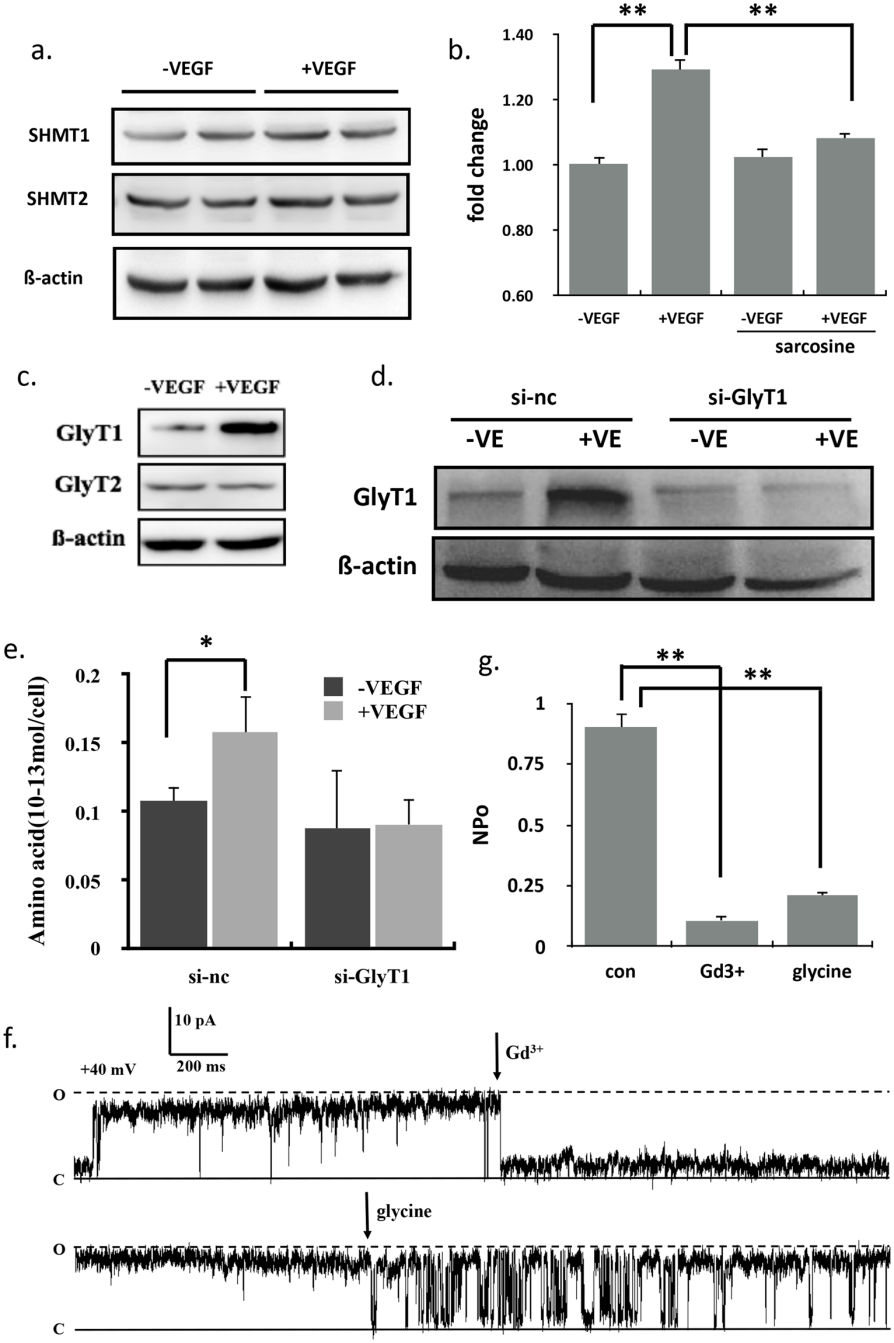
VEGF activated GlyT1 and glycine directly bound to VDAC-1 on the mitochondria outer membrane. (**a**) Immunoblots of SHMT1 and SHMT2 in ECs treated with VEGF (n = 3). (**b**) The effects of sarcosine, an inhibitor of glycine transporter on the proliferation of ECs treated with VEGF (n = 12). (**c**) Immunoblots of GlyT1 and GlyT2 in ECs treated with VEGF (n = 3). (**d**) GlyT1 protein expression by Western blots in HUVECs electroporated with GlyT1 siRNA (si-GlyT1) or control siRNA (si-nc) treated with 20 ng/ml VEGF. (**e**) LC-MS was used to detect relative intracellular glycine in ECs with si-GlyT1 in the absence and presence of VEGF (n = 3). (**f**) VDAC1 was reconstituted in the plasma membrane of Hek-293A. The effects of glycine on single channel currents of VDAC-1 were recorded in +40 mV voltages using single channel patch-clamping (n = 7). (**g**) Statistical analysis of open probability (NPo) in the presence of Gd3 +(a VDAC1 inhibitor) and glycine (n = 7). All values represented mean ± SEM of n independent experiments. **P < 0.01, 1-way ANOVA.

Glycine consumption and expression of the mitochondrial glycine biosynthetic pathway were strongly correlated with the proliferation rate of cancer cells^19^. Defects in glycine metabolism in the mitochondria as a result of reduction in mitochondrial serine hydroxymethyltransferase (SHMT2) and glycine C-acetyltransferase (GCAT) expression are partly responsible for the reduction in mitochondrial translation^20^. To explore the potential relevance of glycine metabolism in VEGF signals, we examined the expression of the mitochondrial glycine synthesis enzyme SHMT2 and plasma glycine synthesis enzyme SHMT1 by the stimulation of VEGF^21^ and found that their expression had no change (Fig. 4a). Sarcosine, the inhibitor of glycine transporter (GlyT) could inhibit the proliferation induced by VEGF (Fig. 4b). There are two kinds of glycine transporters including GlyT1 and GlyT2^22^. VEGF could promote the expression of GlyT1 (Fig. 4c). Knocking down the expression of GlyT1 using siRNA by electroporation (Fig. 4d) and subsequently testing intracellular glycine using LC-MS. VEGF couldn’t increase the intracellular glycine with siGlyT1 treatment (Fig. 4e). Our results suggested that VEGF activated the glycine transporter 1 and increased the intracellular glycine. Incidentally, glycine significantly inhibited the VDAC-1 currents (Fig. 4f and g) as shown in single channel recordings where second messengers are not present; suggesting a novel pathway which glycine regulates mitochondria. Gd^3+^ is a recognized blocker for VDAC-1.

## Discussion

Endothelial cells rely on glycolysis rather than via oxidative phosphorylation^23^. In the current study, VEGF enhanced mitochondrial function, including intracellular ATP production, oxidative respiration, ROS defense enzymes and nitric oxide. Enhanced mitochondrial function was mediated by activating mTOR signaling pathway and the effects relied on the presence of glycine. By LC-MS, glycine showed increasing in intracellular pools by the stimulation of VEGF. Glycine (Gly) is the simplest non-essential amino acid, which participates in the synthesis of proteins and many physiologically important functions, including anti-inflammation^24^, immunomodulation^25^, cytoprotection^26^ and neurotransmitter^27^. Glycine has been showed to be related to the rapid cancer cell proliferation^19^ and could reverse the expression of aging phenotypes^20^. Administration of glycine indeed reduced mild ischemia-reperfusion injury in the kidney *in vivo*, in part by decreasing initial damage and preventing chronic hypoxia^28^. In our study, glycine could mimic the effects of VEGF on ECs to promote *in vitro* matrigel angiogenesis and *in vivo* angiogenesis, including chorioallantoic membrane and hind-limb ischemia. Glycine also effectively protected the mitochondrial functions of ECs. Single channel recordings revealed that glycine could directly bind to VDAC1 and inhibit the opening of VDAC1, which may interfere mitochondrial functions and angiogenesis. There are 3 subtypes of VDAC, but VDAC1 is highly expressed in human, which controls the transit of adenine nucleotides, Ca^2+^, and other metabolites both into and out of the mitochondria^29^. The channel may be also a constituent of the mitochondrial permeability transition pore (PTP) and plays a major role in mitochondria mediated apoptosis^30^. Down regulation of VDAC-1 by siRNA prevents cisplatin induced Bax activation and cell death^31^. Molecular determinant for binding site of glycine on VDAC-1 is required further investigation. What’s more, other amino acids also could promote the mitochondrial function in our results while they weren’t involved in the VEGF pathway. The effects of other amino acids need to be explored further.

In summary, our data provide evidence that VEGF stimulates endothelial cell migration, proliferation and angiogenesis via GlyT1-glycine-mTOR-VDAC1 axis. Our results suggest that glycine is required in VEGF signaling by promoting mitochondrial function via VDAC1 inhibition, which may be of significance to provide new mechanistic insights for therapeutic exploitation. Further investigation of the function of glycine and GlyT1 in angiogenesis may represent novel therapeutic targets for the treatment of angiogenic vascular disorders.

## Materials and Methods

### Cell culture

Human umbilical cords (UC) (n = 4 patients) were collected after a “written informed consent” from all study subject participants with healthy full-term and naturally delivered newborns at the Peking University Third Hospital (Beijing) and the ethics was approved by University Ethics Committee, Institute of Molecular Medicine, Peking University. The experiments were performed in accordance with the guidelines on humane use and care of laboratory animals for biomedical research published by National Institutes of Health (No. 85-23, revised 1996). Human umbilical vein endothelial cells (HUVECs) were freshly isolated from umbilical cord vein. HUVECs were grown in endothelial cell medium(Promocell) and maintained at 37 °C and 5% CO_2_. Experiments were conducted on cells before they reached the fifth passage. HEK-293A was purchased from Cell Library of Chinese Academy of Sciences and cultured (at 37 °C and 5% CO_2_) in the DMEM medium (Promocell, Germany) with 10% FBS (GIBCO, US).

Gd^3+^(Sigma), DCFH-DA (Sigma), rapamycin (Sigma), Sarcosine (Sigma), all amino acids (Sigma).

### Immunoblotting

Cells were lysed ice-cold RIPA lysis buffer (Solarbio) including PMSF (Sigma) and proteinase inhibitor cocktail (25x, Roche). Samples were centrifuged for 15 min at 12000 g at 4 °C and the supernatants were harvested. About 60ug of each protein sample were loaded and separated on 10% Bis-Tris Gel followed by transferring to 0.45 mm PVDF membrane. The membranes were then blocked with 5% non-fat dry milk, probed with appropriate primary antibodies, followed incubation by HRP-conjugated secondary antibodies at a dilution of 1: 5000. The following antibodies were used: rabbit anti-S6 Ribosomal Protein (5G10) (Cell Signaling, #2217), rabbit anti-Phospho-S6 Ribosomal Protein (Ser235/236) (Cell Signaling, #2211),, rabbit anti-mTOR (Proteintech, #20657-1-AP), rabbit anti-SHMT1 (Proteintech, #14149-1-AP), rabbit anti-SHMT2 (Proteintech, #11099-1-AP), mouse anti-ß-actin (Beijing TDY Biotech LTD, #M009), secondary HRP-conjugated anti-mouse and anti-rabbit antibodies (Beijing TDY Biotech LTD, #E009 and #E011 respectively).

### Liquid chromatography–mass spectrometry (LC–MS)

HUVECs were extracted with 1.5 ml of 4:1 v/v MeOH/H_2_O equilibrated at −80 °C. The extract and cells were scraped and collected into 2 ml EP tubes, held at 4 °C for a further 5 min and centrifuged for 5 min at 12,000 g and solvent in the resulting supernatant was evaporated using a speed-vac. Samples were re-suspended in 30 μL HPLC-grade water for mass spectrometry. 8 μL were injected and analyzed using a 5500 QTRAP triple quadrupole mass spectrometer (AB/Sciex) coupled to a Prominence UFLC system (Shimadzu) via selected reaction monitoring (SRM) of 249. Some metabolites were targeted in both positive and negative ion mode for a total of 298 SRM transitions. ESI voltage was + 4900 V in positive ion mode and −4500V in negative ion mode. The dwell time was 5 ms per SRM transition and the total cycle time was 2.09 seconds. Approximately 8–10 data points were acquired for per detected metabolite.

### Mouse model of hindlimb ischemia and evaluation of blood flow

Animal studies carried out in this study were approved and regulated by UK Government Home Office in accordance with the “Guidance on the operation of Animals” (Scientific Procedures) Act, 1986, in agreement with Aston University institutional guidelines and regulations for ethical animal use and care. All aspects of animal use and care took into account NC3RS (replacement, Reduction and Refinement) guidelines. The licensed protocols detailing the regulated animal procedures and care were carried out in accordance to the protocols section 19B of the Home Office licence as approved by UK Government Home Office in accordance with Scientific Procedures Act 1986 and Aston University Research Ethic Committee. Male C57BL/6 (Harlen) (22–26 g) were subjected to unilateral hindlimb surgery under anesthesia with ketamine and Xyaline (100 mg/kg and 10 mg/kg respectively) as previously described^32,33^. The left femoral artery was separated from the vein and nerve, ligated proximally, and injected with 100 ul glycine with the concentration of 140 uM into the saphenous artery. Subsequently vein and artery was ligated and a small (1.5 mm secetion) of vein and artery was removed without effecting the nerve. Prior to tying the artery and vein off and remove. Glycine treatment was continoulsly provided 5% glycine in drinking water. The right hindlimb served as control. Blood flow was measured by Laser Doppler (Moor Instruments, UK). Plantar aspects of the non ischemic and ischemic feet of anesthetized mice (ketamine and Xyaline (100 mg/kg and 10 mg/kg respectively)was measured prior and post surgey in addition to days 3, 7 and 14 days post-surgery. Blood flow values were expressed as the ratio of ischemic to nonischemic hindlimb perfusion.

### *In vitro* angiogenesis assay

HUVECs (1 × 10^4^ cells/well) were seeded in 96 well plates coated with growth factor-reduced Matrigel (#356230, Corning). One hour after seeding, cells were stimulated with either 140 µM glycine or 20 ng/ml VEGF for 5 hours. Formation of capillary-like structures was observed using a Nikon inverted microscope. Images recording and analysis was performed by Image Pro-Plus image analysis software (Media Cybernetics).

### Chorioallantoic angiogenesis membrane assay (CAM)

Fertilized chicken eggs (purchased from China Agriculture University) were incubated for 6 days after breeding at 37 °C with 60% humidity. The method for preparing the CAM was described elsewhere with slight modification^34^. In general, eggs were cleaned with pre-warmed bromo-geramine and a small hole was drilled into the eggshell where the air sac is located. A 1 cm^2^ window was carefully opened for inoculated. The small hole was then vacuumed to exclude air, thus creating space for the CAM. Gelatin sponge was soaked with 140 uM glycine or 20 ng/ml VEGF and seeded on the CAM. The window was then covered with parafilm and the egg was placed back into the incubator. Angiogenesis growth was observed daily. Five days after incubation, CAMs was removed, paraformaldehyde-fixed and observed.

### Measurement of oxygen consumption

The Seahorse Bioscience Extracellular Flux Analyzer (XF24, Seahorse Bioscience Inc., North Billerica, MA, USA) was used to measure OCR (indicative of respiration). HUVECs were seeded (3–5 × 10^4^) into 24 well plate. Appropriate growth media was added and incubated overnight at at 37 °C and 5% O_2_. Cells were first washed and then immersed in unbuffered media prior to incubation in CO_2_ depleted atmosphere for 1 h at at 37 °C. OCR was then measured using a Seahorse Bioscience Extracellular Flux Analyzer under the following parameters as recommended by Seahorse Bioscience guidelines; 8-min cycle of mix (2–4 min), dwell (2 min) and measure (2–4 min).

### Mitochondrial activity assay

For determination of mitochondrial activity, MTT assay was used as reported previously^35^. Briefly, HUVECs were seeded at a density of 2,000 per well into 96-well plates and left to adhere for 12 h. Subsequently, cells were treated with glycine for 5 h, MTT (5 mg/ml, Sigma) was added to each well for 2 h incubation. Aftewards, cells were collected, mixed in DMSO and absorbance measured at 540 nm by Multiskan (Thermo). The relative mitochondrial activity was calculated by the absorbance ratio of the glycine-treated group to the control group.

### ATP measurements

ATP was measured by the CellTiter-Glo^®^ Luminescent Cell Viability Assay. Cells were aliquoted in a white microtitre plate (20,000 cells per well) in a 1:1 ratio with ATP Cell Titer-Glo^®^ Reagent (Promega, Madison, WI, USA). The plate was incubated for 30 min at RT and luminescence was recorded.

### Analysis of mitochondrial transmembrane potential (Δψm)

Quantification of the mitochondrial transmembrane potential was conducted by LASER confocal microscope using the fluorescent probe Rhodamine 123 (R302, Thermo Fisher) to demonstrate the state of mitochondrial transmembrane potential following the manufacturer’s protocol. Briefly, cells were starved with RPMI-1640 without amino acids medium for 50 min and then treat with glycine for 15 min. The cells were incubated with Rh123 (5 μg/ml) in a 37 °C incubator for 20 minutes after washing twice with PBS. To measure specifically the mitochondrial membrane potential (ψ_m_) we quantified the fluorescence intensity before and after applying FCCP, a mitochondrial electron chain uncoupler. The difference of intensity before and after applying FCCP corresponds specifically to the mitochondrial membrane potential.

### Intracellular reactive oxygen species measurement

Quantification of intracellular ROS was conducted by LASER confocal microscope using the fluorescent probe, 2′,7′-dichlorodihydrofluorescein diacetate (DCFH-DA). HUVECs incubated with DCFH-DA (10 μM) in phenol red-free media supplemented for 20 min at 37 °C in the dark. Subsequently HUVECs were washed with PBS prior to quantification with laser confocal microscope.

### Measurements of intracellular nitric oxide

Intracellular nitric oxide (NO) was quantified by laser confocal microscope using the fluorescent probe, DAF-FM DA (Beyotime, S0019). HUVECs were incubated in phenol red-free media supplemented with 5 μM DAF-FM DA in dark for 20 min at 37 °C. They were then washed twice with PBS and immediately observed.

### Electroporation

GlyT1 si-RNA was chemically synthesized and purified by high-performance liquid chromatography (GenePharma, Shanghai, China). The siRNA was transfected using the NeonTM transfection system according to the instructions. Medium 200 (Cat. no. M-200) supplemented with LSGS (Cat. no. S-003-10) was used. The electroporation parameters for HUVECs: pulse voltage was 1350 v; pulse width was 30ms and pulse number was 1.

### Single channel recording

Conventional single channel of the patch-clamp technique was used in the electrophysiological study. Signals were amplified with an Axopatch 200B amplifier (Axon Instruments) and filtered at 1 kHz. Data acquisition and analysis were carried out using pClamp 9.0 (Axon Instruments) software. The extracellular solution was composed of (in mM): 140 NaCl, 5 KCl, 1.8 CaCl_2_, 1 MgCl_2_, 10 HEPES, and 23 sucrose (pH 7.4). The patch pipettes were made of borosilicate glass, and the pipette resistance was 4–6 MΩ with the extracellular solution. We used the extracellular solution in the bath^36^. Single-channel activity was recorded at a pipette voltage of 40 mV. The data were acquired at 20 KHz and low-pass filtered at 5 kHz. During post-analysis, data were further filtered at 200 Hz. Single-channel events were listed and analysed by pclampfit 9.0 (single-channel search-in-analyse function). NPo, the product of the number of channels and the open probability, was used to measure the channel activity within a patch. In total, 50% threshold cross-method was used to determine valid channel openings. Initial (1–2 min) single-channel records were normally used as the control. The activity of VDAC-1 during application of chemicals was normalized to activity during the control period to assess the effects of chemicals on VDAC-1 activity.

### Statistical analysis

Analysis was conducted using either SPSS version 19.0 ((IBM SPSS, Armonk, NY, USA) or Graph Pad Prism (v7.0 USA). All values represent mean ± standard error of the mean. Significant differences between the groups were determined were appropriate by either unpaired one-way analysis of variance test ANOVA or two-way analysis of variance test with repeated measures. A *P*-value P < 0.05 was considered to indicate statistical significant difference.
